# Applications of Neural Networks in Biomedical Data Analysis

**DOI:** 10.3390/biomedicines10071469

**Published:** 2022-06-21

**Authors:** Romano Weiss, Sanaz Karimijafarbigloo, Dirk Roggenbuck, Stefan Rödiger

**Affiliations:** 1 Faculty of Environment and Natural Sciences, Brandenburg University of Technology Cottbus-Senftenberg, Universitätsplatz 1, D-01968 Senftenberg, Germany; romano.weiss@b-tu.de (R.W.); sanaz.karimijafarbigloo@b-tu.de (S.K.); dirk.roggenbuck@b-tu.de (D.R.); 2 Faculty of Health Sciences Brandenburg, Brandenburg University of Technology Cottbus-Senftenberg, D-01968 Senftenberg, Germany

**Keywords:** machine learning, data analysis, neural networks, diagnostics

## Abstract

Neural networks for deep-learning applications, also called artificial neural networks, are important tools in science and industry. While their widespread use was limited because of inadequate hardware in the past, their popularity increased dramatically starting in the early 2000s when it became possible to train increasingly large and complex networks. Today, deep learning is widely used in biomedicine from image analysis to diagnostics. This also includes special topics, such as forensics. In this review, we discuss the latest networks and how they work, with a focus on the analysis of biomedical data, particularly biomarkers in bioimage data. We provide a summary on numerous technical aspects, such as activation functions and frameworks. We also present a data analysis of publications about neural networks to provide a quantitative insight into the use of network types and the number of journals per year to determine the usage in different scientific fields.

## 1. Introduction

Biomedical research creates a myriad of health-related data, which come in different formats, such as numerical (e.g., blood biomarker concentration and gene expression data), time series (e.g., electrocardiogram data) or image data (e.g., histological images and mammograms) [1,2,3]. Bioimage informatics is a subfield of bioinformatics that deals with large-scale, high-throughput computational methods for the analysis of bioimages, particularly cellular and molecular images [4,5].

However, this applies not only to biomedical research but also to other fields, such as forensics [6,7]. The goal is to extract useful knowledge from complicated and heterogeneous images and their associated metadata. Methods and algorithms are often applied to exploit image features corresponding to statistical, geometric and morphological properties. The frequency of image pixels and regions as well as the topological relationship between multiple image objects are also used in this process [4,5,8,9,10,11].

High-content screening and analysis (HCA) encompasses a range of methods for the objectified automated analysis of large image data sets using image processing, computer vision and machine learning. Often, HCA is realised using complex processing pipelines consisting of automated microscopes, automated liquid-handling and cell culture. Typical applications are found in cell biology and drug discovery to identify compounds with pharmacological activity or to identify biomarkers. Researchers use such methods to find specific cell patterns in cells with suitable biomarkers (e.g., tubulin) under a fluorescence microscope [12,13,14,15].

Image processing (including normalisation, segmentation, tracking, spatial transformation, registration and feature calculation) and machine learning are used to automatically process high-dimensional image data into numerical data [16]. From these, biomedical results (e.g., identification of biomarkers, such as differentially regulated RNAs and proteins, breakdown of signalling pathways and recognition of subcellular patterns) can be derived [17,18,19,20,21,22].

Biomarkers or biological markers are objective indicators of a patient’s observable biological state. They are often measured using bodily fluids (blood, urine and lymph) or soft tissue biopsies to determine how well the body responds to a treatment for a disease or medical condition [23]. Analysis of biomarkers on images is usually more difficult compared to other types of data, as the biomarkers are defined not only by numerical values but also by their spatial dependency to each other. An example is the detection of nuclei on an immunofluorescence image [8].

A nucleus is defined not only by pixels that exceed a certain brightness threshold but also by their spatial proximity (bright pixels that are directly next to each other) and the brightness of the surrounding pixels (which has to be lower than those pixels that are inside the nucleus area). Additional detection of biomarkers inside nuclei (e.g., γH2AX or 53BP1 foci; markers for DNA-double strand breaks [15,24]) would not only require their detection (in the case of foci: the detection of areas of high brightness surrounded by areas with a sharp drop of brightness (edges) surrounding them) but also the aforementioned detection of nuclei and the spatial relationship between the detected nuclei and the potential detected biomarkers. Another example would be the detection of cancerous tissue in histological images, which can require certain colour/stain concentrations or specific cellular patterns that need to be recognised.

Whilst these data types can be analysed manually or (semi-)automatically, this approach usually involves a degree of human subjectivity, where classification is usually done by experts. Despite investing a large amount of money in the development of detection approaches, the clinical treatments have not been successful thus far [25]. The main reasons for these problems are the inability of clinicians to obtain sufficient data and perform comprehensive data analysis. In bioimage analysis, a common task is the annotation of certain structures in images (often obtained via techniques, such as microscopy and X-ray tomography or flourescence microscopy) [8,9].

However, the complicated nature of biological images often requires this annotation to be done by a human expert, which is time-consuming, labour-intensive, subjective and may also be error-prone. Due to these limitations of human decision-making, a range of novel software applications has been developed for automatic data classification to assist health care workers. The introduction of machine-learning methods, in particular neural networks, ushered in a new era in this area. For example, researchers in the field of source image forensics are looking at issues of source camera identification, forensics of re-recorded images, computer graphics image forensics, GAN-generated image recognition and source social network identification [6].

One important property of using this type of technology is that machine-learning systems can improve their performance with experience. Three basic paradigms can be distinguished: supervised, semi-supervised and unsupervised learning. As soon as features are extracted and computed, supervised classification methods can be used to recognise different classes of target structures. For example, these can be physiological and pathological phenotypes of cells. Unsupervised clustering can also be suitable to identify new phenotypes. Semi-supervised learning uses both supervised and unsupervised methods [19].

Since their theoretical foundation in 1943, artificial neural networks (ANNs), which mimic the working principle of the human brain, have become an increasingly influential tool for machine learning and data analysis. However, due to hardware limitations, their development in the 20th century was mostly theoretical. With the increase in computer processing power and memory availability, ANNs became a widespread tool for data analysis. From the late 2000s onwards, ANNs were increasingly employed for image classification, resulting in different classification competitions, such as the Pascal VOC challenge (between 2005 and 2012, [26]) and the succeeding ImageNet Large Scale Visual Recognition Challenge (starting 2010, [27]).

Superhuman recognition precision was first achieved by a deep network called DanNet [28] in 2011. Recent attention for the application of deep learning in biomedicine was created by the program AlphaFold [29], which applies neural networks for the prediction of protein folding with unmatched precision in 2022. In particular, the field of bioimage informatics has drastically benefited from the advance of deep learning. Several deep-learning-based analysis approaches have been developed in recent years, ranging from the classification of protein subcellular localisation in immunohistochemistry images [30] to the spatial quantification of clinical biomarker pharmacokinetics [31].

Numerous frameworks for a variety of programming languages can be exploited to use ANNs (Table 1). This includes prominent examples, such as TensorFlow and torch, and lesser known examples, such as scikit-learn (Table 1). Most frameworks are designed to be used without in-depth knowledge of the underlying functionality and concepts. One example of this approach is the TensorFlow Estimator classes, which enable model creation and training without the precise definition of learning methods, network layers and activation functions.

Artificial neural networks have shown their potential particularly for the analysis of image data. For example, forensic scientists use this technology for drowning diagnosis, face verification, image manipulation detection or gunshot wound interpretation [32,33,34,35]. Other researchers in the field of source image forensics are looking at issues of source camera identification, forensics of re-recorded images, computer graphics image forensics, GAN-generated image recognition and source social network identification [6]. Further examples are discussed below for biomedical data.

While this review widely covers the general application of ANN for the analysis of medical data, our focus is the analysis of image data. At this point, we refer to the work of Cynthia Rudin [36]. In her paper, she indicates the problems of these architectures and their problems in the application of biomedical image data. This is because most of the methods we describe here are black boxes whose decision-making process is difficult to comprehend. Instead, she argues for inherently interpretable neural networks with a prototype layer (a prototypical structure, such as the cell nucleus or cytoplasm from, which the network learns a similarity metric between parts of images).

**Table 1:** Selected machine-learning frameworks and their supported programming languages. Note that these frameworks may also have support for Julia, GO, Lisp (programming language by John McCarthy (1958), one of the founding fathers of AI) and Haskell.

Framework	Programming Languages
Tensorflow [37]	Python, R, Java, C++, Go
pyTorch [38]	Python
sklearn [39]	Python
Deeplearning4j [40]	Java
caffe [41]	C++, Python, Matlab
Keras [42]	Python, R
SparkMLlib [43]	Java, Scala, Python, R
Deep Java Library [44]	Java

**Figure 1:**
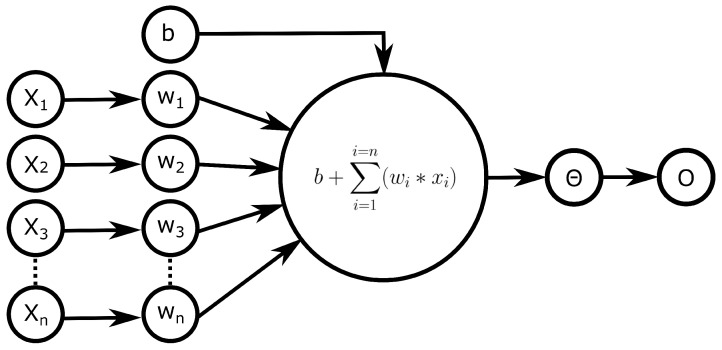
Structure of an artificial neuron. Each neuron receives n weighted inputs (Wn) and a bias (b). First, a weighted sum of each input (X) is calculated. The bias (b) is added to the sum, and the final output/activation (O) is calculated by the activation function (Θ).

The most basic unit of a neural network is a so-called (artificial) neuron (AN), which mimics the function of biological neurons (Figure 1). A neuron is a function with n input parameters and one output value. The output value is calculated as a weighted sum of all input parameters and is passed through a so-called activation function, which modifies the output range of the neuron. Additionally, a bias can be added to shift the activation function on the *x*-axis.

**Figure 2:**
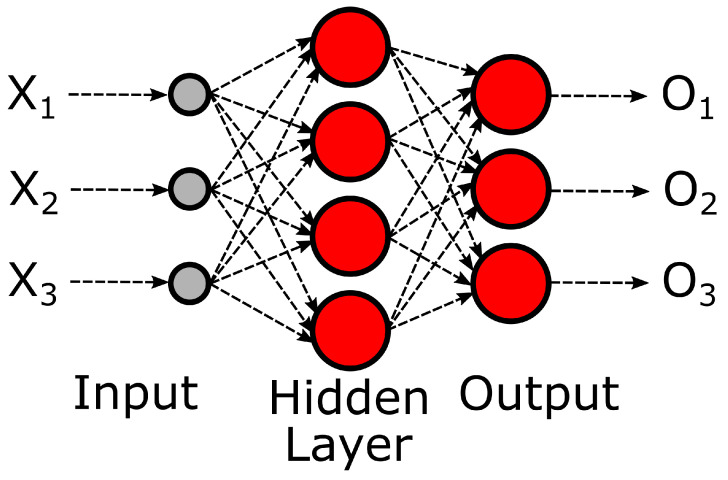
Basic structure of an artificial neural network with three inputs. A neural network consists of artificial neurons, which calculate weighted sums with N input parameters. The output range of an individual neuron will be limited before passing via the application of an activation function. Neurons are arranged in a layered structure, where each neuron receives the activation of all neurons of the previous layer as input parameters. ANNs with more than one layer between the input and output layer (hidden layers) are called deep neural networks. X = Initial input of the network. O = Final output of the network.

The simplest ANNs consist of a layered structure of interconnected ANs to mimic the function of a brain. As each neuron in an ANN receives the activation of all ANs of the previous layer as input parameters, this type of architecture is also called a Dense or Fully Connected Network (Figure 2). Another name would be a Feed-Forward Network, as this architecture only connects consecutive layers. ANNs with more than one layer between the input and output layer (so-called hidden layers) are called deep; thus, training such networks is called deep learning. Training is usually performed via supervised learning (manual annotation of data before training) and adjusting the weights for each input via backpropagation.

Before starting to use deep learning for data analysis, the nature of the data to analyse has to be determined to choose an appropriate network type and architecture. Thereby, the activation functions for each layer have to be chosen carefully to weight between training speed and prediction quality.

## 2. Activation Functions

As stated previously, activation functions are used for limiting the output range of a neuron. Over the last decades, dozens of different activation functions have been proposed for usage in neural networks and deep learning, each with specific advantages and flaws [45] (Table 2).

### 2.1. Early Activation Functions

One of the earliest activation functions was the Heaviside function (named after Oliver Heaviside [46]) also called the unit step function:

(1) $$f\left(x\right)=\left\{\begin{array}{cc} 0 & \mathrm{if}\;x<0\\ 1 & \mathrm{if}\;x\geq 1 \end{array}\right\}$$

This function binarizes the activation by assigning all values below zero to zero and every other value to one. Whilst extremely efficient, the Heaviside function has two severe drawbacks: First, the strict binarization of the activation drastically limits the plasticity of the ANN by limiting the neurons to a similar switch-like function as transistors in electrical circuits. A more critical problem is that learning cannot be performed via backpropagation due to the derivative of the function, which equals 0 for all *x* (except for 0, where the derivative is undefined) [46]. Another, comparably simple approach that solves this problem would be the usage of a linear function (also called an identity function if a equals 1) as an activation function [47], which would perform similarly:

(2) f(x)=a∗x

As the derivative of the function equals a, learning via back propagation would theoretically be possible. However, due to the constant derivative of the function, gradient descent would be input independent and thus convergence of the model, meaning that further training will no longer improve the model inference, cannot be achieved.

### 2.2. Sigmoid Activation Functions

The usage of non-linear functions solves the problem of a constant derivative and thus allows for learning via back propagation. One of the oldest proposed nonlinear function is the sigmoid (s-shaped) *logistic* function:

(3) $$f\left(x\right)=\frac{1}{e^{-x}}$$

It limits the neuron output between 0 and 1 and thus can be useful as an output layer activation for categorisation tasks. Another sigmoid function is the Tangens hyperbolicus (tanh):

(4) $$f\left(x\right)=tanh\left(x\right)=\frac{2}{1+e^{-2x}}-1$$

This function is comparable to the logistic function but limits the output range between −1 and 1. Sigmoid functions suffer from the vanishing gradient problem: As the network becomes deeper, the calculated gradient of the loss function becomes smaller and smaller, reducing the possible weight update and thus impairing the learning of layers closer to the input [48].

### 2.3. Rectified Linear Activation Functions

A solution for the vanishing gradient problem was the introduction of the Rectified Linear Unit function (ReLU):

(5) $$f\left(x\right)=\left\{\begin{array}{cc} 0 & \mathrm{if}\;x<0\\ x & \mathrm{if}\;x\geq 1 \end{array}\right\}$$

This assigns 0 to all values below or equal to zero and otherwise is equal to the identity function [49]. This omits the problem of constant, input-independent learning but introduces a new problem known as “dying ReLu”. Here, neurons have an activation of 0, regardless of input and thus cannot be changed by gradient descent any longer [50]. Different modified forms of ReLU were proposed to address this problem, which all modify the assigned values below 0 to create a non-zero derivative and thus enable gradient descent. Examples are Leaky ReLU:

(6) $$f\left(x\right)=\left\{\begin{array}{cc} x*a & \mathrm{if}\;x<0\\ x & \mathrm{if}\;x\geq 1 \end{array}\right\}$$

which multiplies all values below zero with a small alpha value [51], or ELU:

(7) $$f\left(x\right)=\left\{\begin{array}{cc} a*(e^{x}-1) & \mathrm{if}\;x<0\\ x & \mathrm{if}\;x\geq 1 \end{array}\right\}$$

which shifts function one unit to the left and exponentiates Euler’s number with all values below 0 [52]. The minimal y value of the function can be controlled by variable α. Despite the “dying ReLU” problem, ReLu remains one of the most popular activation functions due to its simple implementation, its fast calculation and the good inference performance [45]. The function is frequently used for convolutional layers [45] but was also proposed to be used as an output function for the last layer [53]. Compared to sigmoid functions, ReLU is far less costly to calculate and appears to be on par in inference quality [54,55].

## 3. Training

However, training efficiency is not only influenced by the selected activation function, the choice of the training algorithm/optimiser is equally important. In general, a backpropagation algorithm attempts to determine the global minimum of the loss function of a neural network. Each training cycle, the network weights are updated, according to their share of network output, to follow the gradient to the local or global minimum of the loss function. Several optimisation algorithms have been developed over time. One of the most prominent algorithms for backpropagation is gradient descent.

### 3.1. Gradient Descent

**Figure 3:**
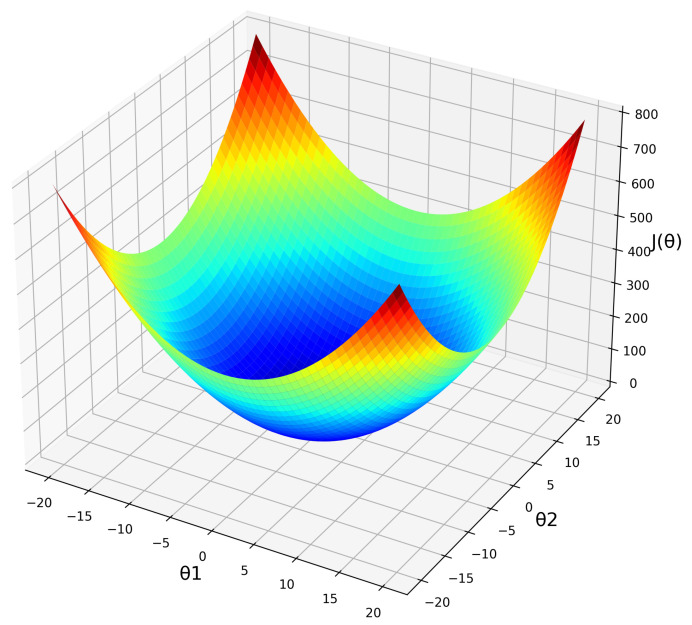
Relationship between the parameter Θ and the objective function J(Θ). The colour gradient indicates high (red) and low (blue) values of J(Θ).

Optimisation algorithms are used to minimise the loss function of a neural network. The most popular optimisers in deep learning are based on gradient descent. Gradient descent, which is a widely used algorithm for optimisation in neural networks, is an iterative optimisation algorithm to minimise the objective function J(Θ) over the training data by updating the parameter Θ [56]. The main idea of gradient descent is to update randomly initialised parameters until the objective function *J* reaches a minimum. Gradient descent was proven to be highly effective in supervised learning [57]. Gradient descent is based on the following process (Figure 3):

- Initiating a random or all-zero vector value to Θ.
- Modification of Θ in order to decrease J(Θ).

To reach the optimal value of Θ, it is recurrently updated until J(Θ) reaches its minimum value. This can be described by the following formula:

(8) $$\Theta^{\left(k+1\right)}=\Theta^{k}-\alpha \nabla J\left(\Theta \right)$$

Here, α is step size or learning rate that indicates the size of the steps to reach a (local) minimum. In gradient descent, we usually normalise the direction of the steepest descent:

(9) $$d^{\left(k\right)}=-\frac{\nabla J(\Theta )}{\left||\nabla J(\Theta )|\right|}$$

Here, *d* is the descent direction and indicates the direction of the steepest descent. The direction of the steepest descent is an guaranteed improvement if the objective function is smooth, the step size is small enough and and the gradient is greater than zero. The direction of the steepest descent is opposite to the direction of gradient ∇J. Thus, to obtain a maximal decrease in *J*, the subsequent direction will always be orthogonal to the current direction [58]. Hence, to have optimal step size α at each step, we have:

(10) $$\alpha^{\left(k\right)}=argminJ\left(\Theta^{\left(k\right)}+\alpha d^{\left(k\right)}\right)$$

Furthermore, to minimise α, we have [58]:

(11) $$\nabla J{\left(\Theta^{\left(k\right)}+\alpha d^{\left(k\right)}\right)}^{T}d^{\left(k\right)}=0$$

(12) $$d^{\left(k+1\right)}=-\frac{\nabla J(\Theta^{\left(k\right)}+\alpha d^{\left(k\right)})}{\left||\nabla J(\Theta^{\left(k\right)}+\alpha d^{\left(k\right)})|\right|}$$

(13) $$d^{(k+1)T}d^{\left(k\right)}=0$$

The above equation indicates that $d^{\left(k+1\right)}$ and $d^{\left(k\right)}$ are orthogonal, which was the condition to have maximum decrease in *J*. Three important variations were developed from gradient descent: *Batch Gradient Descent (BGD)*, *Stochastic Gradient Descent (SGD)* and *Mini BGD*, which differ in the amount of data that are used to calculate the gradient of the objective function. Depending on the amount of data, a trade-off between the accuracy and parameter update time is necessary.

#### 3.1.1. Batch Gradient Descent

Batch Gradient Descent (BGD) performs calculations over the whole training set at each update. As a result, it is very slow on large datasets and is additionally limited by memory capacity. This also introduces redundancy in terms of computation.

(14) $$\Theta^{\left(k+1\right)}=\Theta^{\left(k\right)}-\alpha \nabla J\left(\Theta \right)$$

Batch Gradient Descent is more appropriate for convex or relatively smooth error manifolds and is guaranteed to converge convex error surfaces to the global minimum and non-convex surfaces to local minimums [56,57].

#### 3.1.2. Stochastic Gradient Descent

Stochastic Gradient Descent (SGD) is a widespread algorithm in various machine learning algorithms, e.g., in neural networks and logistic regression. SGD calculates the error and updates the parameters of the model for each training example $x^{\left(i\right)}$ and label $y^{\left(i\right)}$:

(15) $$\Theta^{\left(k+1\right)}=\Theta^{\left(k\right)}-\alpha \nabla J\left(\Theta ;x^{\left(i\right)};y^{\left(i\right)}\right)$$

In contrast to the redundant computations of Batch Gradient Descent, SGD reduces the amount of computations by performing one update at a time, making SGD usually much faster than Batch gradient descent. Despite the faster convergence of SGD, the error function is not as well minimised as for the batch gradient descent. Furthermore, the descent path is noisier, as only one example per update is used. However, this can allow the model to escape from shallow local minima [59].

#### 3.1.3. Mini-Batch Gradient Descent

Mini-Batch Gradient Descent can be seen as the middle ground between the robustness of Stochastic Gradient Descent and the efficiency of Batch Gradient descent, and this is the most common gradient descent algorithm in the field of deep learning. It makes an update for every mini batch of *n* training examples [56]:

(16) $$\Theta^{\left(k+1\right)}=\Theta^{\left(k\right)}-\alpha \nabla J\left(\Theta ;x^{\left(i:i+n\right)};y^{\left(i:i+n\right)}\right)$$

Mini-batch refers to the number of training examples utilised in one iteration, which has a range between 1 and n−1 where *n* is the total dataset size. Finding the appropriate batch size can be challenging: If the size of the batch is chosen small, the learning process converges quickly; however, the descent is noisy, and reaching a minimum is therefore more difficult. Large batch sizes, however, result in a learning process that converges slowly while the error gradient is estimated more accurately. However, some experiments have indicated that a small batch size improves the training stability [60] and takes advantage of speeding up the learning process [61]. The upsides and downsides of variant gradient descent are listed in Table 3.

### 3.2. Optimisation Algorithms

In the following paragraphs, various algorithms are introduced to address the challenges of the three mentioned variants of gradient descent.

#### 3.2.1. Momentum

SGD is a popular optimisation method; however, training takes a long time. Momentum can be used to reduce the training time, particularly when curvatures are high and gradients are either small and noisy or steady [62]. Momentum is a commonly used optimisation algorithm, and many of the latest models are trained with it. It is an adaptive optimisation algorithm that accelerates SGD in the related directions and reduces oscillations [56]. The use of Momentum can be imagined as pushing a ball down a hill. The ball accumulates momentum via gravity and becomes faster. Similarly, gradient causes Momentum, which accumulates in the descent methods. As a result, convergence becomes faster, and oscillation is reduced. Momentum is described by the following Equations [58]:

(17) $$g^{\left(k\right)}=\nabla J\left(\Theta^{\left(k\right)}\right)$$

(18) $$v^{\left(k+1\right)}=\beta v^{\left(k\right)}-\alpha g^{\left(k\right)}$$

(19) $$\Theta^{\left(k+1\right)}=\Theta^{\left(k\right)}+v^{\left(k+1\right)}$$

For β=0, the gradient descent formula is recovered.

#### 3.2.2. Nesterov Accelerated Gradient

**Figure 4:**
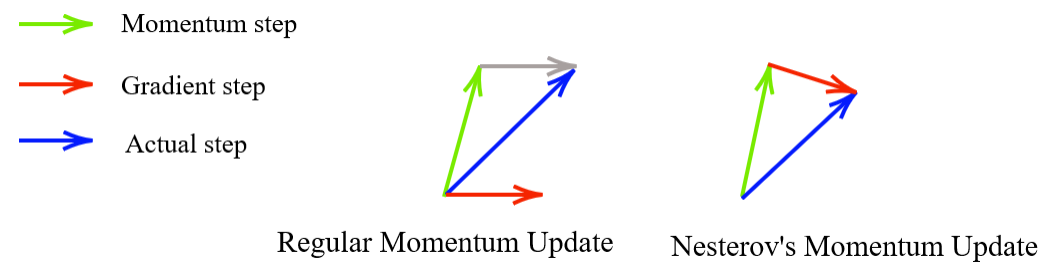
Difference between Nesterov’s Momentum update and the Regular Momentum update.

A problem of Momentum is that it does not slow down sufficiently at the bottom of a valley but rather tends to follow the slope, making it prone to overshooting [58]. Therefore, a more sophisticated optimisation algorithm is needed. Nesterov Accelerated Gradient (NAG) is a slightly different version of Momentum. NGA calculates the point where the current Momentum is pointing to (Figure 4) and thus is able to reduce the step size before the valley slopes up again [56]. The modified Momentum formulas are as follows:

(20) $$v^{\left(k+1\right)}=\beta v^{\left(k\right)}-\alpha \nabla J\left(\Theta^{\left(k\right)}+\beta v^{\left(k\right)}\right)$$

(21) $$\Theta^{\left(k+1\right)}=\Theta^{\left(k\right)}+v^{\left(k+1\right)}$$

A glance at the (Figure 4) reveals the difference between Nesterov’s momentum update and the regular momentum update. The momentum update is performed before calculating the steepest gradient descent vector, which is the main difference between these two. This kind of update refrains from being too fast and ends up increasing responsiveness. As a result, it can enhance the performance of RNN on various tasks, such as the reconstruction of ultrasound images [63].

#### 3.2.3. Adagrad

Unlike Momentum and Nesterov momentum, which update all parameters with the same learning rate, the adaptive subgradient method (Adagrad) uses a different learning rate for each parameter. To adjust the learning rate to the parameters, it considers larger updates for infrequent, and smaller updates for frequent parameters, thus, making it suitable for sparse data. Dean et al. [64] used Adagrad to train large-scale neural nets at Google due to the improved robustness compared to SGD, and it was also utilised to recognise cats in videos. In addition, Adagrad was used to train GloVe, a network to find relations between words [65], due to its ability of much larger updates for infrequent parameters. Adagrad can be described with the following formulas:

(22) $$\Theta_{i}^{\left(k+1\right)}=\Theta_{i}^{\left(k\right)}-\frac{\alpha }{\epsilon +\sqrt{s_{i}^{\left(k\right)}}}g_{i}^{\left(k\right)}$$

(23) $$s_{i}^{\left(k\right)}=\sum_{j=1}^{k}{\left(g_{i}^{\left(j\right)}\right)}^{2}$$

Here, *s* is a vector, and $s_{i}^{\left(k\right)}$ is the sum of the squares of the partials up to time step *k* and respecting Θ. ϵ is a small value (about $1\times 10^{-8})$ to prevent division by zero. Adagrad is less sensitive to the used learning rate (usual value: 0.01); however, a major weakness of the method is the accumulation of the squared gradients in the denominator. Every added term is positive, and thus the accumulated sum continues increasing during the training. As a result, the learning rate often becomes infinitesimally small before convergence. It was shown that AdaGrad can have fewer generalisation errors compared to the Adam optimiser [66].

#### 3.2.4. Adadelta

Adaptive Delta (Adadelta) is a more robust extension of Adagrad. The aim of Adadelta is to deal with the monotonically decreasing learning rate of Adagrad based on restricting the window of accumulated past gradients to some fixed size *w* [67] rather than accumulating all past gradients.

(24) $$\Theta_{i}^{\left(k+1\right)}=\Theta_{i}^{\left(k\right)}-\frac{RMS(\Delta \Theta_{i})}{\epsilon +RMS(g_{i})}g_{i}^{\left(k\right)}$$

Since the value of RMS(ΔΘ) is not known, it is estimated with the Root Mean Square (RMS) of parameter updates until the previous time step [60]. As can be seen, it is unnecessary to specify a default learning rate, since it has been eliminated from the update rule.

#### 3.2.5. Root Mean Square Propagation

The Root Mean Square Propagation (RMS Prop), similar to Momentum, is a technique to speed up gradient descent [68]. It maintains an exponentially decaying average of squared gradients and divides the learning rate by the root of this average [56]. The average is updated according to:

(25) $${\hat{s}}^{\left(k+1\right)}=\gamma {\hat{s}}^{\left(k\right)}+\left(1-\gamma \right)\left(g^{\left(k\right)}⊙g^{\left(k\right)}\right)$$

(26) $$\Theta_{i}^{\left(k+1\right)}=\Theta_{i}^{\left(k\right)}-\frac{\alpha }{\epsilon +\sqrt{{\hat{s}}_{i}^{\left(k\right)}}}g_{i}^{\left(k\right)}$$

(27) $$\Theta_{i}^{\left(k+1\right)}=\Theta_{i}^{\left(k\right)}-\frac{\alpha }{\epsilon +RMS(g_{i})}g_{i}^{\left(k\right)}$$

where the decay γ∈[0,1] was suggested by Tieleman and Hinton [68] to be set to 0.9, while a good default value for the learning rate α is 0.001.

#### 3.2.6. Adam

The adaptive moment estimation method (Adam) adapts learning rates to each parameter. Both exponentially decaying squared gradient $s^{\left(k+1\right)}$, such as RMSProp and Adadelta, and exponentially decaying gradients $v^{\left(k+1\right)}$, such as momentum, are stored in the Adam algorithm. $v^{\left(k+1\right)}$ and $s^{\left(k+1\right)}$ are estimates of first-order momentum and second-order momentum of the gradients, respectively [69]. To introduce a bias, $v^{\left(k+1\right)}$ and $s^{\left(k+1\right)}$ are initialised to zero during the initial time steps and particularly when the decay rates are small. The mathematical notation for Adam can be expressed as following:

(28) $$v^{\left(k+1\right)}=\gamma_{v}v^{\left(k\right)}+\left(1-\gamma_{v}\right)g^{\left(k\right)}$$

(29) $$s^{\left(k+1\right)}=\gamma_{s}s^{\left(k\right)}+\left(1-\gamma_{s}\right)\left(g^{\left(k\right)}⊙g^{\left(k\right)}\right)$$

(30) $${\hat{v}}^{\left(k+1\right)}=\frac{v^{\left(k+1\right)}}{1-\gamma_{v}^{k}}$$

(31) $${\hat{s}}^{k+1}=\frac{s^{\left(k+1\right)}}{1-\gamma_{s}^{k}}$$

(32) $$\Theta^{\left(k+1\right)}=\Theta^{\left(k\right)}-\frac{\alpha {\hat{v}}^{\left(k+1\right)}}{\epsilon +\sqrt{{\hat{s}}^{\left(k+1\right)}}}$$

According to a publication of Kingma and Ba [70], good starting values are α=0.001, $\gamma_{v}=0.9,\;\gamma_{s}=0.999$ and $\epsilon =1\times 10^{-8}$. Adam is an efficient method for computation, uses less memory for implementation and is invariant to diagonal rescaling of the gradients. Thus, it is suitable for huge data sets, noisy data, inadequate gradients and non-stationary problems that require small tuning [62].

#### 3.2.7. AdaMax

AdaMax is a variant of Adam, which was changed based on the use of infinity norm ($u_{t}$). The Adam update rule for weights is based on scaling the gradients inversely proportional to a $l_{2}$ norm of the past and current gradients. The $l_{2}$ norm based update rule can be generalised to a $l_{p}$ norm based update rule. In the case of using large p values, norms become numerically unstable, while, for $l_{\infty }$, a stable algorithm appears [70]. The AdaMax algorithm can be described by the following formulas:

(33) $$s^{\left(k+1\right)}=\gamma_{s}s^{\left(k\right)}+\left(1-\gamma_{s}\right){\left|g^{\left(k\right)}\right|}^{2}$$

(34) $$s^{\left(k+1\right)}=\gamma_{s}^{p}s^{\left(k\right)}+\left(1-\gamma_{s}^{p}\right){\left|g^{\left(k\right)}\right|}^{p}$$

(35) $$u^{\left(k+1\right)}=\gamma_{s}^{\infty }s^{\left(k\right)}+\left(1-\gamma_{s}^{\infty }\right){\left|g^{\left(k\right)}\right|}^{\infty }$$

(36) $$u^{\left(k+1\right)}=\max(\gamma_{s}\cdot s^{\left(k\right)},|g^{\left(k\right)}\left|\right)$$

by replacing $\epsilon +\sqrt{{\hat{s}}^{\left(k+1\right)}}$ in the Adam equation with $u^{\left(k+1\right)}$, the AdaMax update rule is obtained:

(37) $$\Theta^{\left(k+1\right)}=\Theta^{\left(k\right)}-\frac{\alpha {\hat{v}}^{\left(k+1\right)}}{u^{\left(k+1\right)}}$$

### 3.3. Choosing the Right Optimiser

The choice of the right optimiser is highly dependent on the dataset to analyse. If the input data are sparse, then good results can be achieved using one of the adaptive learning-rate methods, such as Adam or Adadelta. Additionally, using adaptive-learning methods eliminates the need to fine-tune the learning rate to obtain optimal results. However, it should be considered that they are computationally costly, since they calculate and keep all the past gradients and their squares to update the next parameters.

Furthermore, the adaptive-learning optimisers converge to different minima points in comparison with fixed learning-rate optimisers. Both RMSprop and Adadelta are extensions of Adagrad, which overcome Adagrad’s monotonically decreasing learning rate. The difference between both methods lies in the usage of the RMS of parameter updates in the numerator update rule.

The Adam algorithm extends RMSprop by adapting learning rates to each parameter and adding bias-correction and momentum. The Adam technique can be utilised in the case of high-dimensional parameters and huge data sets. RMSprop, Adadelta and Adam are algorithms with similar behaviour and can perform well under comparable conditions. However, Kingma and Ba [70] indicate that its bias-correction results in better performance in Adam compared to RMSprop towards the end of optimisation as gradients become sparser. Therefore, Adam might be the best of the presented choices.

Interestingly, recent work found that SGD can produce better results when combined with a good learning rate annealing schedule. Although SGD is usually able to find a minimum, it might take considerably longer than other optimisers. It also depends much more on a robust initialisation and choice of learning rate. Moreover, its fluctuation helps avoid local minima; however, it may become stuck in saddle points. In summary, when a good learning rate schedule is required, SGD with momentum can be a viable choice. In the case of searching for fast convergence and training a complex neural network, one of the adaptive learning rate methods should be chosen.

### 3.4. Back Propagation

Backpropagation, presented in 1986 by Rumelhart and McClelland [71], is a short form of “backward propagation of errors”. It is a common method of training neural networks and an iterative gradient descent training procedure. It utilises the loss function and gradient descent method to modify the parameters (called weights) of a network. The backpropagation process can be described as follows [72]:

*Forward propagation*: Input is entered into the network and propagated from the input layer, via the hidden layer, to the output layer. Input values are multiplied with weights of connecting nodes, and values of hidden layer nodes are obtained. The weight and offset value of the network are kept constant during the forward propagation.

*Back propagation*: In the case that there is a difference between the expected output and the achieved output, the error of the network is propagated from the output layer to the input layer. The network carries on updating the weights until the error becomes minimal. Updating weights is performed from the output layer and hidden layer and can be described via the following formulas [73,74]:

The error function is defined as follows

(38) $$e_{j}\left(n\right)=d_{j}\left(n\right)-y_{j}\left(n\right)$$

(39) $$E\left(n\right)=\frac{1}{2}\sum e_{j}^{2}\left(n\right)$$

(40) $$E_{AV}=\frac{1}{N}\sum_{n=1}^{N}E(n)$$

Here, $e_{j}\left(n\right)$ is the error of the *j*th neuron, E(n) is the instantaneous error energy, $E_{AV}$ is the averaged squared error energy, and *N* is the total number of training data. Furthermore, $d_{j}\left(n\right)$ and $y_{j}\left(n\right)$ are the expected output and the obtained output of the *j*th neuron, respectively. In the following, the way of obtaining output of the layers is explained:

(41) $$y_{j}\left(n\right)=\phi_{j}\left(v_{j}\left(n\right)\right)$$

(42) $$v_{j}=\sum_{i=0,...,m}w_{ji}x_{i}$$

Here, $w_{ji}$ indicates the weight of the connection from the *i*th neuron to the *j*th neuron, *m* is the number of neurons, $x_{i}$ is the input signal of *i*th neuron, and ϕ is a function. The methods of minimising the loss function and updating the weights are as follows:

(43) $$\frac{\partial E(n)}{\partial w_{ji}\left(n\right)}=\frac{\partial E(n)}{\partial e_{j}\left(n\right)}\frac{\partial e_{j}\left(n\right)}{\partial y_{j}\left(n\right)}\frac{\partial y_{j}\left(n\right)}{\partial v_{j}\left(n\right)}\frac{\partial v_{j}\left(n\right)}{\partial w_{ji}\left(n\right)}$$

(44) $$\frac{\partial E(n)}{\partial w_{ji}\left(n\right)}=-e_{j}\left(n\right)\phi^{\prime }\left(v_{j}\left(n\right)\right)x_{i}\left(n\right)$$

The weight correction Δw is obtained as follows:

(45) $$\Delta w_{ji}\left(n\right)=-\alpha \frac{\partial E(n)}{\partial w_{ji}\left(n\right)}=\alpha e_{j}\left(n\right)\phi^{\prime }\left(v_{j}\left(n\right)\right)x_{i}\left(n\right)$$

The local gradient $\delta_{j}$ of the *j*th neuron is computed using the chain rule, which can be seen as follows:

(46) $$\delta_{j}\left(n\right)=-\frac{\partial E(n)}{\partial v_{j}\left(n\right)}=-\frac{\partial E(n)}{\partial e_{j}\left(n\right)}\frac{\partial e_{j}\left(n\right)}{\partial y_{j}\left(n\right)}\frac{\partial y_{j}\left(n\right)}{\partial v_{j}\left(n\right)}$$

(47) $$\delta_{j}\left(n\right)=e_{j}\left(n\right)\phi^{\prime }\left(v_{j}\left(n\right)\right)$$

(48) $$\Delta w_{ji}\left(n\right)=\alpha \delta_{j}\left(n\right)x_{i}\left(n\right)$$

Here, α is the learning rate. Ultimately, the weight is updated as follows:

(49) $$w_{ji}\left(n+1\right)=w_{ji}\left(n\right)+\Delta w_{ji}\left(n\right)$$

To update weights from the hidden layer, errors are propagated from hidden layer down to the input layer. The method of calculating a local gradient is different, and it is computed as follows:

(50) $$\delta_{j}\left(n\right)=-\frac{\partial E(n)}{\partial y_{j}\left(n\right)}\frac{\partial y_{j}\left(n\right)}{\partial v_{j}\left(n\right)}$$

(51) $$\frac{\partial E(n)}{\partial y_{j}\left(n\right)}=\sum e_{k}\frac{\partial e_{k}\left(n\right)}{\partial y_{j}\left(n\right)}$$

(52) $$\frac{\partial E(n)}{\partial y_{j}\left(n\right)}=\sum e_{k}\frac{\partial e_{k}\left(n\right)}{\partial v_{k}\left(n\right)}\frac{\partial v_{k}\left(n\right)}{\partial y_{j}\left(n\right)}$$

in order to compute $\frac{\partial e_{k}\left(n\right)}{\partial v_{k}\left(n\right)}$, remember:

(53) $$e_{k}\left(n\right)=d_{k}\left(n\right)-y_{k}\left(n\right)=d_{k}\left(n\right)-\phi_{k}\left(v_{k}\left(n\right)\right)$$

(54) $$\frac{\partial E(n)}{\partial y_{j}\left(n\right)}=-\sum e_{k}\phi^{\prime }\left(v_{k}\left(n\right)\right)w_{kj}\left(n\right)$$

(55) $$\frac{\partial E(n)}{\partial y_{j}\left(n\right)}=-\sum_{k}\delta_{k}\left(n\right)w_{kj}\left(n\right)$$

(56) $$\delta_{j}\left(n\right)=-\frac{\partial E(n)}{\partial y_{j}\left(n\right)}\frac{\partial y_{j}\left(n\right)}{\partial v_{j}\left(n\right)}$$

(57) $$\delta_{j}\left(n\right)=\phi_{j}^{\prime }\left(v_{j}\left(n\right)\right)\sum_{k}\delta_{k}\left(n\right)w_{kj}\left(n\right)$$

Finally, the weight is updated as shown in following formulas:

(58) $$\Delta w_{ji}\left(n\right)=\alpha \delta_{j}\left(n\right)x_{i}\left(n\right)$$

(59) $$w_{ji}\left(n+1\right)=w_{ji}\left(n\right)+\Delta w_{ji}\left(n\right)$$

## 4. Potential Training Problems

The automatic approach of neural network training has several potential pitfalls, which can negatively impact training and overall classification performance or even completely invalidate the classification. First, problems can arise due to the initialisation of the network weights. An intuitive approach would be to simply initialise all values and biases with a fixed value, e.g., zero. This, however, impairs learning as such initialised neurons tend to develop similar weights [75]. A solution to this problem was proposed by random value initialisation—the application of randomly changing the learning rate [75,76]. Further, training problems can arise when an inappropriate learning rate is selected: too small and the training progress per training cycle is small, while too large and minima of the loss function might not be achievable.

Another potential point of failure is the training used data set and training time. Data sets should have a sufficient size and be diverse enough to fully represent the target data, otherwise the network might not be able to generalise enough to perform well under real testing conditions. Another error regarding the dataset is to break the strict separation of the training and the validation data set, which usually results in a gross overestimation of network performance.

Regarding the training time, two phenomenons can be seen: underfitting and overfitting. Underfitting occurs when the model’s training time is too short to properly adapt to the dataset, whilst overfitting occurs when a network is trained too extensively and thus loses its capability to generalise. Both under- and overfitting result in poor network performance. However, whilst underfitting can be easily spotted by predictions accuracy during training, overfitting can easily be overlooked if no adequate validation dataset is used.

A more sinister problem is called Clever Hans Predictors (CHP). These kinds of neural networks seemingly perform well under laboratory conditions, making them difficult to spot before deployment. The problem of CHP arises if a network focuses on features that are logically irrelevant for inference [77]. These could be watermarks or other text on images or on background details instead of the objects of interest. This problem becomes particularly serious when only certain classes contain irrelevant characteristics. If both the training and validation dataset are flawed, the only way to spot CHPs is via a review of its activation for specific samples, which both takes time and requires a certain degree of expertise for both the used dataset and programming.

Recent examples of flawed applications of neural networks in the health sector were described in the studies of Wynants et al. [78] and Roberts et al. [79], where they analysed prediction models that had been recently described to support COVID-19 diagnosis (Wynant et al.: 232 models; Roberts et al.: 62 models). Both studies did not recommend any of the studied models for clinical use because all of them had at least one or more of the above listed problems. This highlights the urgency of good datasets and network design.

## 5. Network Types

Different types of network architectures have been developed over time to address different types of data and problems. The first developed types were fully connected neural networks, followed by convolutional neural networks. Currently, more complicated networks, such as U-Nets or Generative Adversarial Neural Networks are also abundant.

### 5.1. Convolutional and Generative Adversarial Neural Networks

Convolutional Neural Networks (CNNs) are specialised ANNs that are designed to solve pattern recognition tasks via machine learning. Thereby, rather than receiving scalar input, as with dense networks, CNNs receive matrix input, such as images. The basis for modern CNNs was laid by the neocognitron by Fukushima in 1980 (43) and the time delay neural networks by Waibel in 1987 [80]. One of the first widely recognised networks was LeNet, a CNN for the recognition of postal zip codes, designed by LeCun et al. in 1989 [81]. CNNs are composed of three main components: convolutional, downsampling/pooling and dense layers.

In contrast to dense layers, convolutional layers perform convolution, which means each neuron calculates weighted sums of a predefined set of inputs for each input rather than forming a weighted sum for all inputs. The size and weighting of the area is defined by a convolution kernel, which is shared between all neurons of a layer. This allows convolutional layers to perform image processing tasks, such as edge and corner detection. Per convolutional layer, multiple convolution kernels are trained to perform different processing tasks.

**Figure 5:**
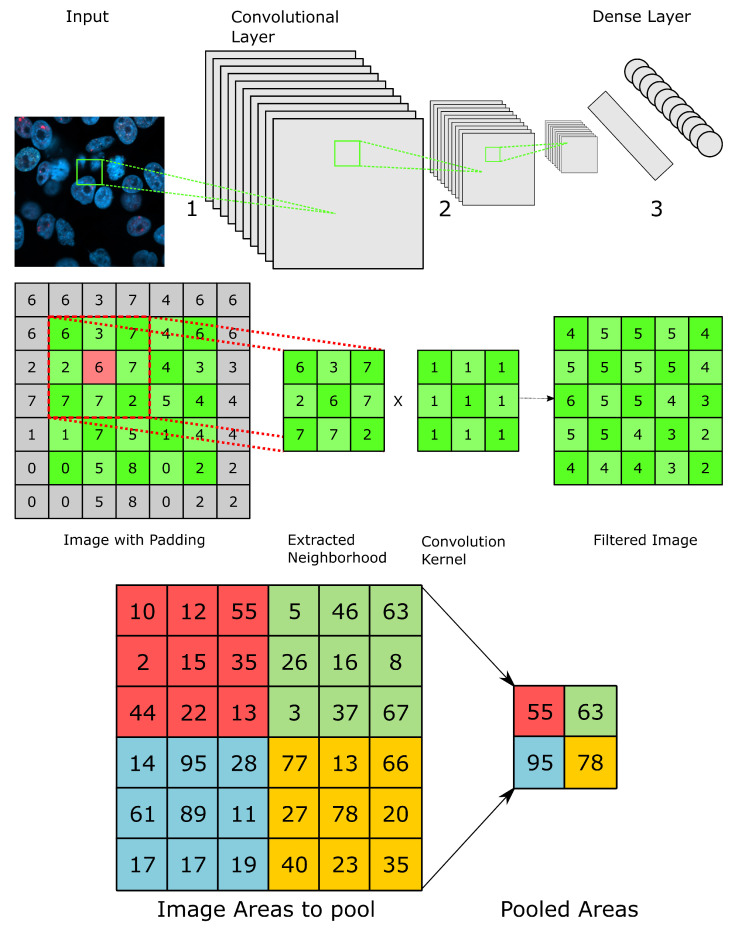
General structure and functioning of a convolutional neural network (CNN). (**Top**): Architecture of a CNN. A CNN usually contains a various number of convolutional layers, which are each directly followed by pooling/downsampling layers to both reduce the network complexity and abstract the input. The output of the last maximum pooling layer is flattened at the transition to the dense layer. 1: Convolution; 2: Convolution and pooling; and 3: Linearisation. (**Middle**): Depiction of the process of convolution Padding is indicated in gray. (**Bottom**): Depiction of Maximum Pooling with a radius of 1.5 for both axes. Pooling is performed by determining the maximum of each region (shown in different colours).

To reduce the input dimensionality as well as to abstract it, each convolutional layer is followed by a downsampling layer. Whilst different methods for pooling are available, the most commonly used is maximum pooling, where the maximum of the specified area is used as the output. In addition, reducing the output dimension of a convolutional layer and thus subsequently the complexity of the network, it can also help to prevent overfitting by reducing the availability of raw input information. To be compatible with the dense part of the network, the output of the last downsampling layer is vectorised before passing. The subsequent processing is then performed as described for ANNs (Figure 5).

Whilst CNNs are useful for whole image classification, their ability for image segmentation is limited: Due to their dense layer, a convolutional network can output a certainty if an object is contained in an image but not where the object is located. Another limitation is the detection of multiple different objects in the same image. One approach to overcome this hurdle was the introduction of regional CNNs (R-CNNs). R-CNNs are designed as described for regular CNNs but are fed with overlapping segments of the image, which are classified individually, allowing to create a heatmap of object locations.

A further advantage for image segmentation was the introduction of fully convolutional networks (FCNs), first described by Long et al. in 2014 [82]. Contrary to CNNs, FCNs are composed completely out of convolutional and pooling layers. The dense part of a CNN is replaced by one upsampling layer to match the output and input dimensions. This design gives FCNs several advantages over CNNs. Due to the missing dense layers and the shared convolution kernels of convolutional layers, FCN architecture allows for dynamic, arbitrary input sizes. Furthermore, as the output of an FCN is a matrix rather than a vector, pixelwise image segmentation can be achieved.

Another direction to deal with mentioned problems is superpixel segmentation. A Superpixel can be defined as a group of pixels that perceptually shares common characteristics while considering spatial constraints. Superpixels carry more information compared to pixels and also provide a compact representation of an image, which is useful for reducing computational complexity [83]. They are becoming increasingly popular in many computer vision and image processing algorithms, such as image segmentation, semantic labelling, object detection and tracking.

Gheshlaghi et al. [84] used the superpixel segmentation technique to overcome dimensionality problems for multiple sclerosis lesion detection. Fang et al. [85] proposed a superpixel segmentation algorithm to segment two-dimensional bone images and three-dimensional brain images. In this paper, the blocks with the same features were merged and to segment the superpixel/voxel medical image, the final distance with the intensity feature and the location feature, and the gradient feature was considered.

The FCN architecture was further refined by the introduction of U-Nets by Ronneberger et al. in 2015 [86]. A U-Net can be divided into two different sections: downsampling and upsampling. As with a normal FCN, the downsampling part of the network is composed of alternating convolutional and pooling layers. The upsampling of a U-Net is composed of transposed convolutional layers, whereby the number of transposed convolutional layers matches the number of pooling layers.

Furthermore, the upsampling rate is set to match the downsampling rate so that the input and output shape are equal. To further improve the segmentation quality, each upsampling layer is connected to a downsampling block; thereby, the first downsampling block is connected to the last upsampling layer, the second downsampling layer is connected to the second last upsampling layer and so on. This allows deeper layers to use low-level data, which helps to improve the predictions.

In comparison to the older dense neural network, neural networks with convolutional layers have different advantages: Due to the weight sharing, convolutional layers can process much more input parameters. The performed convolution also allows each neuron to consider local neighbourhood parameter relationships rather than weighing each parameter individually, which allows the network to recognise features, such as edges, corners and patterns. Additionally, the added pooling forces the network to become less reliant on the input data as part of it is cut at each pooling layer, which might help the network to generalise better.

A major drawback of convolutional neural networks is their dependence on huge datasets for training, as they are neither rotation-, translation- nor color-invariant. This requires multiple images of the object of interest in different positions and rotations as well as lighting conditions. This could partially be alleviated by the introduction of random rotation/translation into the dataset (data augmentation) but might introduce difficult to detect artificial artefacts, which could negatively influence the classification results.

A special use case of FCNs/CNNs are Generative Adversarial Networks (GANs), which can be used to create images and other output data comparable to its training data from noise [87,88]. This is achieved by training an FCN and CNN simultaneously, where one network is designed to receive noise (FCN, called the generator) and output fabricated images, whilst the second network is trained to differentiate between the fabricated and real input images (CNN, called the discriminator).

The generator is generally trained using the inference difference of the discriminator between generated and real images. This allows the generator to train unsupervised. However, compared to the training of other types networks, GANs are notoriously difficult to train as they require hyperparameter fine tuning for both the generator and the discriminator: Both overly high and overly low inference quality of the discriminator can cause poor generation quality. Several different approaches have been proposed to address this problem, such as noise addition for the discriminator input or “freezing” of the lower layers of a pretrained discriminator [89,90].

### 5.2. Recurrent Neural Networks

Recurrent neural networks (RNNs) are a special class of neural networks that are derived from feed-forward networks and possess the ability to process sequential data. This is accomplished by saving previous states of the network in specialised recurrent units (RUs), which are organised in loop-like structures. These RUs can be regarded as dense layers with tanh activation, which save their respective activation as a hidden state. This hidden state is passed between and modified by each RU for inference.

Whilst this, in theory, allows RNNs to learn long-term dependencies, they suffer in practice from either vanishing or exploding gradients during training, drastically limiting the inference performance of classical RNNs [91]. This problem was addressed by the development of Long Short-Term Memory Networks (LSTMs) [91,92]. Each standard LSTM unit has two states: the cell state, which is solely passed between the different LSTM cells and the hidden state, which constitutes the cell output. These states are influenced by three different gates: The forget-gate, the modification-gate and the output-gate.

The cell state is first influenced by the forget-gate, which weighs the previous cell state. In other words, the forget-gate decides which information of the cell state is relevant for further inference. The second gate is the modification gate, which modifies the cell state according to the cells learned parameters. Both gates depend on the hidden state of the previous cell. The last gate is the output gate, which further modifies the cell state, dependent on the previous hidden state, to create this cell’s hidden state.

Since the first introduction of LSTMs, different modifications have been proposed. Peephole LSTMs [93], for example, interconnect the gates with the cell state and thus changes the information flow inside each unit. A more radical change was proposed with Gated Recurrent Units (GRUs) [94], which replaces the forget and the modification gate with a singular update gate and merges the cell and hidden state.

Due to their structure, RNNs cannot be trained via regular backpropagation. Instead, training is usually performed via backpropagation through time (BPTT), a variant of backpropagation, which unravels the RNN and applies gradient descent using accumulation of the error for each recurrent unit of the network. Regardless of their challenges in training convergence, they exhibit remarkable performance in practical application. As an example, in one application [95], a combination of CNN feature extraction and LSTM classification was used to classify histopathological images.

The images were divided into patches to alleviate the limitation of the computational requirements, and then the CNN model was utilised to embed each path into a latent space (e.g., feature vectors). Next, the feature description of all patches was concatenated to form an input sequence for the LSTM model. Finally, with the sequence-to-sequence transformation capability of the LSTM model, the contextual dependency among patches was modelled to produce an image-level prediction. R. Azad et al. [96] further developed the LSTM idea into a segmentation model to unify the feature description driving from the encoder–decoder module in a non-linear fashion.

### 5.3. Graph Neural Networks

Graph-like data has been proven to be difficult to analyse using network architectures, such as CNNs or RNNs, which expect a predefined set of input features. To tackle this kind of data, a new kind of architecture was developed, named Graph Neural Network (GNN, [97]). A GNN is usually used to tackle the following tasks: Node classification and regression; edge classification and prediction; and graph classification, regression and matching [98].

In general, a GNN usually consists of three main modules: a propagation module, used to aggregate information from neighbouring nodes, a sampling module, working in conjunction with the propagation module, and a pooling module, which reduces the data complexity [98]. An improvement of a GNN is the so-called Graph Convolutional Network (GCN), which applies convolutional layers to extract information [99,100]. The concept of convolution was applied to GNN to decrease the high computational weight of the previous GNN design. In CNNs, convolution operates on local Euclidean structure, while in GCNs, it operates on non-Euclidean data (e.g., graph) to incorporate irregular data structure [101]. The main difference between GNNs and dense neural networks is the graph transfer function. More specifically, similar to the dense neural network, the graph transfer module learns the full connection weights between all nodes; however, in addition, it considers the importance of the edge connections.

This might explain why the GCN network is more capable of learning structural information shared among all nodes and is more prone to missing data points. It should also be noted that the GCN model requires precise data structuring (unlike CNN that works on the raw data), which might limit the applicability of this architecture in different applications. Time and space complexity are also other limitations of the GCN network. Particularly, the backpropagation operation in the GCN [22] networks requires saving all computed nodes along with the intermediate states, which requires high computational memory specifically for the large graph.

In addition, as stated before, the GCN is a generalised form of CNN architectures and requires more training time to capture the underlying representation. Yu et al. [102] used Graph Neural Networks for the determination of biomarkers from microarray data. In this work, first, the graph structure was constructed using the gene interaction network, and then the Graph Neural Network was used for the link prediction method to enhance the graph structure data. Li et al. [103] proposed a novel biomarker selection method in microarray data by Combining Graph Neural Networks and Gene Relationships. In this paper, Graph Neural Networks were used to select features and characterise node information. Then, a spectral clustering method was applied to filter redundant features.

### 5.4. Transformers

Transformers are a neural network architecture that was proposed in the year 2017 by Vaswani et al. [104]. This architecture is built around attention-driven blocks, which are neural network layers that aggregate information from the whole input sequence [105]. The model was originally designed for machine translation by modelling long-range dependencies and multi-head attention mechanisms (the proposed model of Vaswani et al. is one the most famous in the field of Natural Language Processing (NLP)) but is currently also increasingly used for image processing. Although Convolutional Neural Networks (CNNs) have been the most popular deep neural networks in medical image analysis, they are weak in learning long-range data because of their localised receptive field [106].

Application of the long range learning capabilities for computer vision tasks was made possible by the development of Vision Transformers (proposed by Dosovitskiy et al., 2020 [107]). In this model architecture, an image is transformed into a sequence of non-overlapped patches, where each patch indicates a spatial location on the input image. Next, by applying the multi-head attention mechanism followed by the Multilayer Perceptron (MLP) module, it learns the importance of each sequence component to model object-level recognition. An extension of this architecture (e.g., [108]) was further proposed to alleviate the problem of weak global representation stemming from the local nature of the CNN modules.

From another perspective, the lack of global representation in the CNN model usually pushes the learning strategy of this network toward the texture clues, which weakens the shape-based description. The Transformer network utilises the attention mechanism on top of the image patches to model global representation. Hence, it has the potential in learning global and, consequently, shape-based information.

In addition, the sequence-to-sequence learning strategy deployed in the Transformer model empowers this architecture to better model inductive bias compared to the CNN counterparts. However, with a higher number of parameters, the quadratic computational complexity of the attention operation and hunger for the large training data are among the main drawbacks of this network architecture.

As an example of utilising Transformers in clinical applications, Lum et al. [109] proposed an attention-based video model to detect the disease signatures and learn clinically relevant imaging biomarkers. A knowledge transfer approach was also used to overcome the problem of data limitations. In another application [110], a Transformer-based method was utilised to reconstruct Electrocardiography’s (ECG) signal from the photoplethysmography (PPG) version. More specifically, a multi-head attention mechanism was designed to perform sequence to sequence prediction processes using waveform data. The predicted ECG signal along with the PPG version was then used to monitor cardiovascular diseases.

### 5.5. Challenges of Neural Networks

Despite their advantages, neural networks also have to contend with some difficulties. The most glaring problem of neural networks is their extremely high complexity, which makes it difficult to explain the results obtained (also called black-box networks). In recent years, some efforts have been made to make neural networks more interpretable (e.g., through reverse engineering or various visualisation techniques, [111,112]); however, as neural networks grow in size and complexity, the problem is likely to become worse. This is problematic because black-box networks can hide other problems, such as CHP (Section 4) and dataset bias, i.e., the uneven distribution of available data for different social groups, which can lead to incorrect diagnoses and/or treatments for women and minority groups when such networks are used as tools by clinicians [113,114].

## 6. Usage of Neural Networks for Medical Data Analysis

The appropriate architecture for analysis largely depends on the type of data. We differentiate between four data types: scalar data, n×n matrices (images), series data and graph data.

### 6.1. Scalar Data

Scalar data are the most basic type of data obtained during diagnosis. Typical medical data with scalar characteristics are:

- Heart rate.
- Blood pressure.
- Blood type.
- Glucose level.
- Spectroscopic measurements.
- Biomarker concentration.

A dense network usually receives and outputs scalar data, making it useful for classification and clustering. The main application of dense networks lies in diagnosis assistance for medical personnel. Networks trained with different biomarkers as input and the respective medical diagnosis as output could be utilised particularly for the diagnosis of rare and thus often overlooked diseases. The applicability of such diagnosis models has been evaluated for acute nephritis, abnormal cardiac behaviour, carcinoma, valve stenosis and other diseases [115,116].

Dense networks were also used for the estimation of skin parameters for the reconstruction of 2D maps of blood volume fraction and blood oxygen saturation in the skin, the measurement of oxygen saturation and haemoglobin concentration in living tissue, the differentiation of smokers from non-smokers and the discrimination of human bodies from bones and teeth remains [117,118,119,120].

### 6.2. Images

Several diagnostic procedures produce image data. Some typical examples are:

- MRI/CT images.
- Tissue section images.
- Immunofluorescence images.
- Retinal images.

In addition, there are applications in forensics that deal with, for example, the identification of image sources, forensic face verification in videos, drowning diagnosis or the interpretation of gunshot wounds [32,33,34,35]. The input for CNNs/FCNs/U-Nets is an n-dimensional matrix; it can thus be used for classification and clustering of image data. Since their development, CNNs, FCNs/U-Nets and derivatives thereof have been used extensively for classification of image-based medical data. Examples can be found, among others, for the detection of Alzheimer’s Disease, brain tumours, lung cancer, liver cancer and mitosis and nuclear atypia detection for breast cancer [121,122,123,124,125]. GANs also have seen various uses in medical data analysis from the estimation of CT images from MR images, the detection of brain lesions and retinal vessel detection to image synthesis for recognition network training [126,127,128,129].

### 6.3. Series Data

Series data are a special case of data compared to the previously described ones. Instead of only analysing one data point for classification, the network needs to draw conclusions from a series of ordered data points. These data points can be scalar data points as well as image data. Typical series data are:

- Biomarker concentration over time.
- ECG.
- Live cell imaging.

RNNs with memory cells (LSTMs and GRU) are a suitable choice to tackle this kind of data. They have been used for sepsis detection, survival prediction for heart transplantation, hospital readmission rate prediction for lupus patients or MRI image reconstruction [130,131,132,133,134].

### 6.4. Graph Data

A graph describes the characteristics of and relationships between data points. Typical series data are:

- Protein/molecule structures.
- Patient data.

Graphs are difficult to analyse using classical neural networks because of their lack of predefined structure. The network types to analyse graph data are GNNs and derivatives, such as GCNs. GCNs have been utilised in many fields, including computer vision applications, person re-identification, action localisation and also in medical image analysis. These networks were successfully used, e.g., for diagnosis prediction, prescription prediction and biomarker identification [135,136,137]. Zhang et al. [128] considered the supervoxels from the brain MRI volume as the nodes of the graph and used GCN to classify supervoxels into different types of tissues. Zhou et al. [101] utilized a GCN for the grading of colorectal cancer, and Shi et al. [138] used a GCN to classify cervical cells.

## 7. Publication Development between 2000 and 2021

To analyse the usage of different neural network architectures, we searched the PubMed API (https://pubmed.ncbi.nlm.nih.gov/, accessed on 27 January 2022) to find articles with publication dates between 2000 and 2021 that matched a respective keyword (Table 4). Overall, 42,335 publications were analysed. First, the PubMed IDs of matching publications were fetched using the *esearch* interface. A document summary, containing among others, information about the authors and journal, was then requested for each ID using the *efetch* interface.

**Figure 6:**
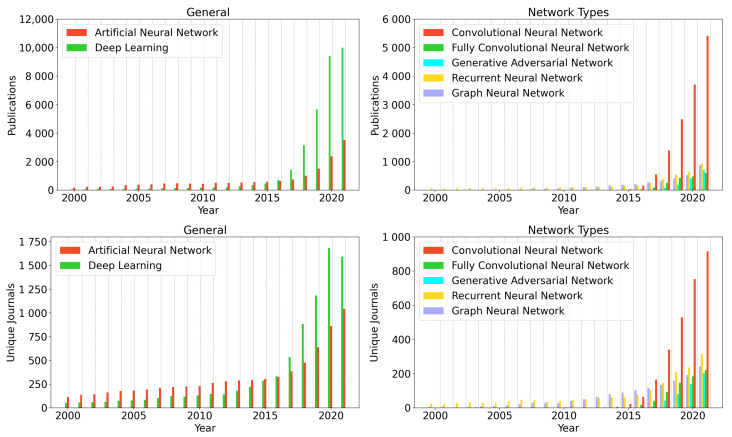
(**Top**): Number of published articles per year. (**Bottom**): Number of journals that published articles matching the following keywords: Artificial Neural Network, Deep Learning, Convolutional Neural Network, Fully Convolutional Neural Network, Generative Adversarial Network, Recurrent Neural Network and Graph Neural Network. Articles were fetched using the PubMed API (https://www.ncbi.nlm.nih.gov/home/develop/api/, accessed on 27 January 2022).

For each keyword, the number of published articles, and journals between 2000 and 2021 was analysed (52,940 publications in total, publications without a full journal name were excluded). Thereby, for all searched keywords, an increase in the number published articles and a respective increase in publishing journals was observed (Figure 6). The largest increase in publications per year was observable for the broad terms “Artificial Neural Network” (N = 185 (2000) to N = 3531 (2021) and “Deep Learning” (N = 62 (2000) to N = 10,000 (2021)), which are generally broadly used to indicate the usage of neural networks.

For specific architectures, an increase in publications per year for all architecture types were observed, particularly after the year 2015. This might be explained by ever-increasing computing prowess over the last twenty years, the emergence of Graphics Processing Units (GPU; described as early as 2005 [139])—and cloud computing driven machine learning (ML) as well as increasing popularity due to easy access ML APIs, such as Tensorflow or Torch. Thereby, the largest number of publications was observed for Convolutional Neural Networks with 5415 publications in 2021, followed by Recurrent Neural Networks (N = 926) and Graph Neural Networks (N = 872).

The least number of peak publications was found for Fully Convolutional Neural Networks (N = 608). The high number of papers incorporating convolutional neural networks can be explained by their ability to categorise multidimensional data as well as their easier implementation compared to generative or recurrent networks. Whilst approaches have been shown to analyse image data for both recurrent and generative networks, their implementation is usually more complicated, and more parameter fine-tuning is required for successful training.

The number of unique journals in general shows a similar trend as the number of publications for all searched keywords. The largest number of unique journals was observed for the keyword Deep Learning (N = 1591 (2021)), followed by Artificial Neural Network (N = 1042 (2021)) and Convolutional Neural Network (N = 915 (2021)). The least diverse publication fields were observed for Graph Neural Network (N = 242 (2021)), Fully Convolutional Neural Networks (N = 221 (2021)) and Generative Adversarial Neural Networks (N = 204 (2021)).

**Figure 7:**
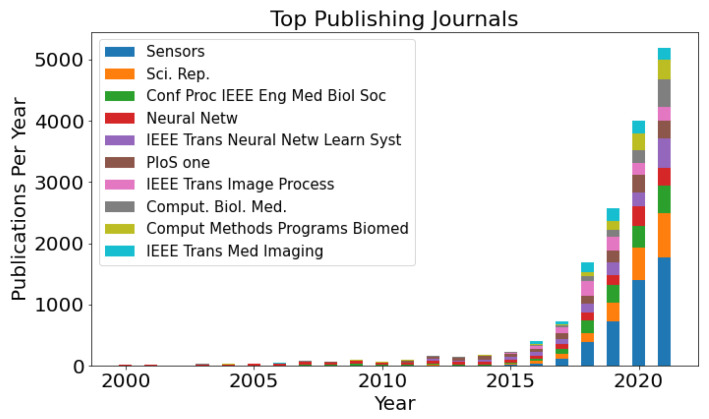
The top 10 journals between 2000 and 2021 with the overall most publications. Interestingly, in the last five years, journals with a bioanalytical scope (*Sensors* and *Scientific Reports* (Sci. Rep.) and *PLOS One* (PloS one)) had a high share.

Additionally, the ten journals with the overall most publications between 2000 and 2021 were investigated (Figure 7). The overall most publications were published in the journal Sensors (Basel, Switzerland) with 4502 publications, followed by Scientific reports (1867 publications) and Conf Proc IEEE Eng Med Biol Soc (1570 publications).

## Figures and Tables

**Table 2:** Commonly used activation functions. The graphs for each function (red) and its derivative (blue) are shown.

Name	Function [Range]	Function (Red) and Derivative (Blue)
linear	f(x)=a∗x Range:[−b,b]	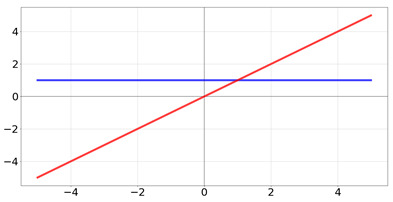
Heaviside	$$f\left(x\right)=\left\{\begin{array}{cc} 0 & \mathrm{if}\;x<0\\ 1 & \mathrm{if}\;x\geq 1 \end{array}\right\}$$ Range:[−∞,∞]	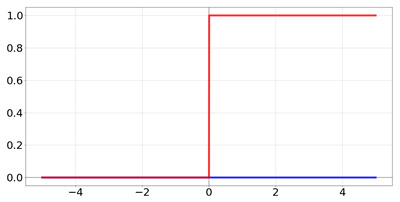
Rectified Linear Unit	$$f\left(x\right)=\left\{\begin{array}{cc} 0 & \mathrm{if}\;x<0\\ x & \mathrm{if}\;x\geq 1 \end{array}\right\}$$ Range:[0,∞]	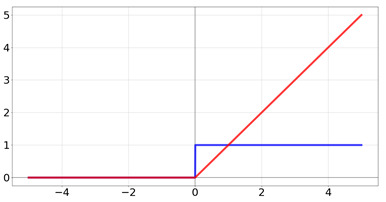
Logistic/sigmoid	$f\left(x\right)=\frac{1}{1+e^{-x}}$ Range:[0,1]	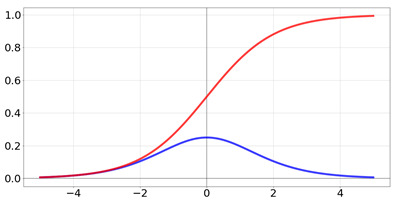
tanh	$f\left(x\right)=tanh\left(x\right)=\frac{2}{1+e^{-2x}}-1$ Range:[−1,1]	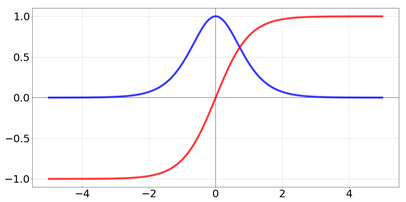
elu	$$f\left(x\right)=\left\{\begin{array}{cc} a*(e^{x}-1) & \mathrm{if}\;x<0\\ x & \mathrm{if}\;x\geq 1 \end{array}\right\}$$ Range:[−∞,∞]	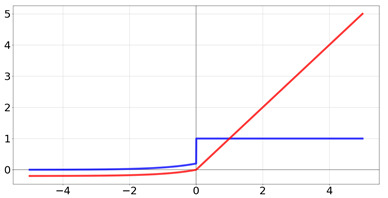

**Table 3:** Comparison of gradient descent algorithm variations and their advantages and disadvantages.

Algorithm	Advantages	Disadvantages
Batch Gradient Descent	The reduced update frequency leads to a more stable error gradient and better convergence on some problems. Given sufficient time for convergence gives an optimal solution.	Cannot escape shallow local minima easily. Can be very slow and do not fit in memory for large datasets. Performs redundant computations for large datasets.
Stochastic Gradient Descent	The increased model update frequency can result in faster learning and convergence. The noisy update process can enable the model to avoid local minima. It is faster and requires fewer computations than BGD.	The frequent updates cause a noisy gradient signal that may cause the model parameters and error to jump around. The noisy learning process can make it hard for the algorithm to settle on a minimum error. The model gives a good but not optimal solution.
Mini-Batch Gradient Descent	The higher update frequency of this model, compared to batch gradient descent, provides a more robust convergence, and it helps avoid local minima. Higher computational efficiency compared to SGD. Less memory usage than other methods	To calculate error of the model, it is required to accumulate error information across mini-batches, such as BGD.

**Table 4:** Used search terms for performed queries. A query using the listed search terms (separated by “;”) for each architecture type was performed.

Architecture	Search Terms
General	Artificial Neural Network; Deep Learning
Convolutional Neural Networks	Convolutional Neural Network; Fully Convolutional Neural Network
Generative Adversarial Neural Networks	Generative Adversarial Neural Network
Recurrent Neural Networks	Recurrent Neural Network
Graph Neural Networks	Graph Neural Networks

## Data Availability

Not applicable.

## Abbreviations

The following abbreviations are used in this manuscript:

ADAM	Adaptive Moment Estimation Method
AdaDelta	Adaptive Delta
Adagrad	Adaptive Subgradient Method
ANN	Artificial Neural Network
AI	Artificial Intelligence
API	Application Programming Interface
Backpropagation	Backward Propagation of Error
BGD	Batch Gradient Descent
BPTT	Backpropagation Through Time
CHP	Clever Hans Predictors
CNN	Convolutional Neural Networks
CT	Computed Tomography
COVID-19	Coronavirus Disease 2019
ECG	Electrocardiography
FCN	Fully Convolutional Network
GCN	Graph Convolutional Network
GNN	Graph Neural Network
GRU	Gated Recurrent Unit
GPU	Graphics Processing Unit
HCA	High-Content Analysis
ID	Identifier
LSTM	Long Short-Term Memory Network
Mini BGD	Mini Batch Gradient Descent
ML	Machine Learning
MRI	Magnetic Resonance Imaging
MLP	Multilayer Perceptron
NAG	Nesterov Accelerated Gradient
NLP	Natural Language Processing
PPG	Photoplethysmography
R-CNN	Regional Convolutional Neural Network
ReLU	Rectified Linear Unit function
RMS	Root Mean Square
RMSprop	Root Mean Square Propagation
RNN	Recurrent Neural Network
RU	Recurrent Unit
SGD	Stochastic Gradient Descent
tanh	Tangens Hyperbolicus
U-Net	Architecture for Semantic Segmentation

## References

[1] Zafeiris D., Rutella S., Ball G.R. (2018). An Artificial Neural Network Integrated Pipeline for Biomarker Discovery Using Alzheimer’s Disease as a Case Study. Comput. Struct. Biotechnol. J..

[2] Diao J.A., Kohane I.S., Manrai A.K. (2018). Biomedical informatics and machine learning for clinical genomics. Hum. Mol. Genet..

[3] Min S., Lee B., Yoon S. (2017). Deep learning in bioinformatics. Brief. Bioinform..

[4] Coelho L.P., Glory-Afshar E., Kangas J., Quinn S., Shariff A., Murphy R.F., Hutchison D., Kanade T., Kittler J., Kleinberg J.M., Mattern F., Mitchell J.C., Naor M., Nierstrasz O., Pandu Rangan C., Steffen B. (2010). Principles of Bioimage Informatics: Focus on Machine Learning of Cell Patterns. Linking Literature, Information, and Knowledge for Biology.

[5] Peng H. (2008). Bioimage informatics: A new area of engineering biology. Bioinformatics.

[6] Yang P., Baracchi D., Ni R., Zhao Y., Argenti F., Piva A. (2020). A Survey of Deep Learning-Based Source Image Forensics. J. Imaging.

[7] Thurzo A., Kosnáčová H.S., Kurilová V., Kosmeľ S., Beňuš R., Moravanský N., Kováč P., Kuracinová K.M., Palkovič M., Varga I. (2021). Use of Advanced Artificial Intelligence in Forensic Medicine, Forensic Anthropology and Clinical Anatomy. Healthcare.

[8] Schneider J., Weiss R., Ruhe M., Jung T., Roggenbuck D., Stohwasser R., Schierack P., Rödiger S. (2019). Open source bioimage informatics tools for the analysis of DNA damage and associated biomarkers. J. Lab. Precis. Med..

[9] Meijering E., Carpenter A.E., Peng H., Hamprecht F.A., Olivo-Marin J.C. (2016). Imagining the future of bioimage analysis. Nat. Biotechnol..

[10] Chessel A. (2017). An Overview of data science uses in bioimage informatics. Methods.

[11] Cardona A., Tomancak P. (2012). Current challenges in open-source bioimage informatics. Nat. Methods.

[12] Rödiger S., Schierack P., Böhm A., Nitschke J., Berger I., Frömmel U., Schmidt C., Ruhland M., Schimke I., Roggenbuck D. (2013). A highly versatile microscope imaging technology platform for the multiplex real-time detection of biomolecules and autoimmune antibodies. Adv. Biochem. Eng..

[13] Willitzki A., Hiemann R., Peters V., Sack U., Schierack P., Rödiger S., Anderer U., Conrad K., Bogdanos D.P., Reinhold D. (2012). New platform technology for comprehensive serological diagnostics of autoimmune diseases. Clin. Dev. Immunol..

[14] Sowa M., Großmann K., Scholz J., Röber N., Rödiger S., Schierack P., Conrad K., Roggenbuck D., Hiemann R. (2015). The CytoBead assay—A novel approach of multiparametric autoantibody analysis in the diagnostics of systemic autoimmune diseases. J. Lab. Med..

[15] Reddig A., Rübe C.E., Rödiger S., Schierack P., Reinhold D., Roggenbuck D. (2018). DNA damage assessment and potential applications in laboratory diagnostics and precision medicine. J. Lab. Precis. Med..

[16] Stack E.C., Wang C., Roman K.A., Hoyt C.C. (2014). Multiplexed immunohistochemistry, imaging, and quantitation: A review, with an assessment of Tyramide signal amplification, multispectral imaging and multiplex analysis. Methods.

[17] Feng J., Lin J., Zhang P., Yang S., Sa Y., Feng Y. (2017). A novel automatic quantification method for high-content screening analysis of DNA double strand-break response. Sci. Rep..

[18] Millard B.L., Niepel M., Menden M.P., Muhlich J.L., Sorger P.K. (2011). Adaptive informatics for multifactorial and high-content biological data. Nat. Methods.

[19] Shariff A., Kangas J., Coelho L.P., Quinn S., Murphy R.F. (2010). Automated Image Analysis for High-Content Screening and Analysis. J. Biomol. Screen..

[20] Caie P.D., Walls R.E., Ingleston-Orme A., Daya S., Houslay T., Eagle R., Roberts M.E., Carragher N.O. (2010). High-Content Phenotypic Profiling of Drug Response Signatures across Distinct Cancer Cells. Mol. Cancer Ther..

[21] Lu J., Tsourkas A. (2009). Imaging individual microRNAs in single mammalian cells in situ. Nucleic Acids Res..

[22] Carragher N.O., Brunton V.G., Frame M.C. (2012). Combining imaging and pathway profiling: An alternative approach to cancer drug discovery. Drug Discov. Today.

[23] Strimbu K., Tavel J.A. (2010). What are biomarkers?. Curr. Opin. HIV AIDS.

[24] Ruhe M., Rabe D., Jurischka C., Schröder J., Schierack P., Deckert P.M., Rödiger S. (2019). Molecular biomarkers of DNA damage in diffuse large-cell lymphoma—A review. J. Lab. Precis. Med..

[25] Rabbi F., Dabbagh S.R., Angin P., Yetisen A.K., Tasoglu S. (2022). Deep Learning-Enabled Technologies for Bioimage Analysis. Micromachines.

[26] Everingham M., Eslami S.M.A., Van Gool L., Williams C.K.I., Winn J., Zisserman A. (2015). The Pascal Visual Object Classes Challenge: A Retrospective. Int. J. Comput. Vis..

[27] Russakovsky O., Deng J., Su H., Krause J., Satheesh S., Ma S., Huang Z., Karpathy A., Khosla A., Bernstein M. (2015). ImageNet Large Scale Visual Recognition Challenge. Int. J. Comput. Vis..

[28] Ciresan D.C., Meier U., Masci J., Gambardella L.M., Schmidhuber J. Flexible, High Performance Convolutional Neural Networks for Image Classification. Proceedings of the Twenty-Second International Joint Conference on Artificial Intelligence.

[29] Senior A.W., Evans R., Jumper J., Kirkpatrick J., Sifre L., Green T., Qin C., Žídek A., Nelson A.W.R., Bridgland A. (2020). Improved protein structure prediction using potentials from deep learning. Nature.

[30] Xue Z.Z., Wu Y., Gao Q.Z., Zhao L., Xu Y.Y. (2020). Automated classification of protein subcellular localization in immunohistochemistry images to reveal biomarkers in colon cancer. BMC Bioinform..

[31] Hoffmann B., Gerst R., Cseresnyés Z., Foo W., Sommerfeld O., Press A.T., Bauer M., Figge M.T. (2022). Spatial quantification of clinical biomarker pharmacokinetics through deep learning-based segmentation and signal-oriented analysis of MSOT data. Photoacoustics.

[32] Oura P., Junno A., Junno J.A. (2021). Deep learning in forensic gunshot wound interpretation—A proof-of-concept study. Int. J. Leg. Med..

[33] Zeng J., Zeng J., Qiu X. Deep learning based forensic face verification in videos. Proceedings of the 2017 International Conference on Progress in Informatics and Computing (PIC).

[34] Homma N., Zhang X., Qureshi A., Konno T., Kawasumi Y., Usui A., Funayama M., Bukovsky I., Ichiji K., Sugita N. A Deep Learning Aided Drowning Diagnosis for Forensic Investigations using Post-Mortem Lung CT Images. Proceedings of the 2020 42nd Annual International Conference of the IEEE Engineering in Medicine & Biology Society (EMBC).

[35] Bayar B., Stamm M.C. (2016). A Deep Learning Approach to Universal Image Manipulation Detection Using a New Convolutional Layer. Proceedings of the 4th ACM Workshop on Information Hiding and Multimedia Security.

[36] Rudin C. (2019). Stop explaining black box machine learning models for high stakes decisions and use interpretable models instead. Nat. Mach. Intell..

[37] Abadi M., Agarwal A., Barham P., Brevdo E., Chen Z., Citro C., Corrado G.S., Davis A., Dean J., Devin M. TensorFlow: Large-Scale Machine Learning on Heterogeneous Systems. Proceedings of the 12th USENIX conference on Operating Systems Design and Implementation (OSDI’16).

[38] Paszke A., Gross S., Massa F., Lerer A., Bradbury J., Chanan G., Killeen T., Lin Z., Gimelshein N., Antiga L., Wallach H., Larochelle H., Beygelzimer A., Alché-Buc F.D., Fox E., Garnett R. (2019). PyTorch: An Imperative Style, High-Performance Deep Learning Library. Advances in Neural Information Processing Systems 32.

[39] Pedregosa F., Varoquaux G., Gramfort A., Michel V., Thirion B., Grisel O., Blondel M., Prettenhofer P., Weiss R., Dubourg V. (2011). Scikit-learn: Machine Learning in Python. J. Mach. Learn. Res..

[40] Eclipse Deeplearning4J. https://github.com/eclipse/deeplearning4j.

[41] Jia Y., Shelhamer E., Donahue J., Karayev S., Long J., Girshick R., Guadarrama S., Darrell T. (2014). Caffe: Convolutional Architecture for Fast Feature Embedding. Proceedings of the 22nd ACM International Conference on Multimedia.

[42] Chollet F. Keras. https://github.com/fchollet/keras.

[43] Meng X., Bradley J., Yavuz B., Sparks E., Venkataraman S., Liu D., Freeman J., Tsai D., Amde M., Owen S. (2016). MLlib: Machine Learning in Apache Spark. J. Mach. Learn. Res..

[44] Deep Java Library (DJL). https://github.com/deepjavalibrary/djl.

[45] Nwankpa C., Ijomah W., Gachagan A., Marshall S. (2018). Activation Functions: Comparison of trends in Practice and Research for Deep Learning. arXiv.

[46] Legua M.P., Morales I., Sánchez Ruiz L.M., Gervasi O., Murgante B., Laganà A., Taniar D., Mun Y., Gavrilova M.L. (2008). The Heaviside Step Function and MATLAB. Computational Science and Its Applications—ICCSA 2008.

[47] Lederer J. (2021). Activation Functions in Artificial Neural Networks: A Systematic Overview. arXiv.

[48] Roodschild M., Gotay Sardiñas J., Will A. (2020). A new approach for the vanishing gradient problem on sigmoid activation. Prog. Artif. Intell..

[49] Xu B., Wang N., Chen T., Li M. (2015). Empirical Evaluation of Rectified Activations in Convolutional Network. arXiv.

[50] Lu L. (2020). Dying ReLU and Initialization: Theory and Numerical Examples. Commun. Comput. Phys..

[51] Maas A.L. (2013). Rectifier Nonlinearities Improve Neural Network Acoustic Models. Proc. ICML.

[52] Clevert D.A., Unterthiner T., Hochreiter S. (2016). Fast and Accurate Deep Network Learning by Exponential Linear Units (ELUs). arXiv.

[53] Agarap A.F. (2019). Deep Learning using Rectified Linear Units (ReLU). arXiv.

[54] Jain A., Jain V., Bansal P., Tushir M., Balas V.E., Srivastava R. (2021). Effect of Activation Functions on Deep Learning Algorithms Performance for IMDB Movie Review Analysis. Proceedings of the International Conference on Artificial Intelligence and Applications.

[55] Lau M.M., Lim K.H. Review of Adaptive Activation Function in Deep Neural Network. Proceedings of the 2018 IEEE-EMBS Conference on Biomedical Engineering and Sciences (IECBES).

[56] Ruder S. (2017). An overview of gradient descent optimization algorithms. arXiv.

[57] Zhou X. (2018). Understanding the Convolutional Neural Networks with Gradient Descent and Backpropagation. J. Phys. Conf. Ser..

[58] Kochenderfer M.J., Wheeler T.A. (2019). Algorithms for Optimization.

[59] Ketkar N. (2017). Deep Learning with Python: A Hands-On Introduction.

[60] Masters D., Luschi C. (2018). Revisiting Small Batch Training for Deep Neural Networks. arXiv.

[61] Bengio Y., Montavon G., Orr G.B., Müller K.R. (2012). Practical Recommendations for Gradient-Based Training of Deep Architectures. Neural Networks: Tricks of the Trade.

[62] Yaqub M., Feng J., Zia M., Arshid K., Jia K., Rehman Z., Mehmood A. (2020). State-of-the-Art CNN Optimizer for Brain Tumor Segmentation in Magnetic Resonance Images. Brain Sci..

[63] Wang H., Dalkilic B., Gemmeke H., Hopp T., Hesser J. Ultrasound Image Reconstruction Using Nesterov’s Accelerated Gradient. Proceedings of the 2018 IEEE Nuclear Science Symposium and Medical Imaging Conference Proceedings (NSS/MIC).

[64] Dean J., Corrado G., Monga R., Chen K., Devin M., Mao M., Ranzato M.a., Senior A., Tucker P., Yang K. Large Scale Distributed Deep Networks. Proceedings of the Advances in Neural Information Processing Systems, Harrah’s and Harveys.

[65] Pennington J., Socher R., Manning C. (2014). Glove: Global Vectors for Word Representation. Proceedings of the 2014 Conference on Empirical Methods in Natural Language Processing (EMNLP).

[66] Fang J.K., Fong C.M., Yang P., Hung C.K., Lu W.L., Chang C.W. AdaGrad Gradient Descent Method for AI Image Management. Proceedings of the 2020 IEEE International Conference on Consumer Electronics-Taiwan (ICCE-Taiwan).

[67] Alfian G., Syafrudin M., Ijaz M., Syaekhoni M., Fitriyani N., Rhee J. (2018). A Personalized Healthcare Monitoring System for Diabetic Patients by Utilizing BLE-Based Sensors and Real-Time Data Processing. Sensors.

[68] Tieleman T., Hinton G. (2012). Lecture 6.5-rmsprop: Divide the Gradient by a Running Average of Its Recent Magnitude.

[69] Fei Z., Wu Z., Xiao Y., Ma J., He W. (2020). A new short-arc fitting method with high precision using Adam optimization algorithm. Optik.

[70] Kingma D.P., Ba J. (2017). Adam: A Method for Stochastic Optimization. arXiv.

[71] Paola J.D., Schowengerdt R.A. (1995). A review and analysis of backpropagation neural networks for classification of remotely-sensed multi-spectral imagery. Int. J. Remote Sens..

[72] Li J., Cheng J.h., Shi J.y., Huang F., Jin D., Lin S. (2012). Brief Introduction of Back Propagation (BP) Neural Network Algorithm and Its Improvement. Advances in Computer Science and Information Engineering.

[73] Kim P. (2017). MATLAB Deep Learning: With Machine Learning, Neural Networks and Artificial Intelligence.

[74] Czum J.M. (2020). Dive Into Deep Learning. J. Am. Coll. Radiol..

[75] Thimm G., Fiesler E. (1997). High-order and multilayer perceptron initialization. IEEE Trans. Neural Netw..

[76] Masood S., Chandra P. (2012). Training neural network with zero weight initialization. Proceedings of the CUBE International Information Technology Conference on CUBE ’12.

[77] Lapuschkin S., Wäldchen S., Binder A., Montavon G., Samek W., Müller K.R. (2019). Unmasking Clever Hans predictors and assessing what machines really learn. Nat. Commun..

[78] Wynants L., Van Calster B., Collins G.S., Riley R.D., Heinze G., Schuit E., Bonten M.M.J., Dahly D.L., Damen J.A., Debray T.P.A. (2020). Prediction models for diagnosis and prognosis of COVID-19: Systematic review and critical appraisal. BMJ.

[79] Roberts M., Driggs D., Thorpe M., Gilbey J., Yeung M., Ursprung S., Aviles-Rivero A.I., Etmann C., McCague C., AIX-COVNET (2021). Common pitfalls and recommendations for using machine learning to detect and prognosticate for COVID-19 using chest radiographs and CT scans. Nat. Mach. Intell..

[80] Waibel A., Hanazawa T., Hinton G., Shikano K., Lang K. (1989). Phoneme recognition using time-delay neural networks. IEEE Trans. Acoust. Speech Signal Process..

[81] LeCun Y., Boser B., Denker J.S., Henderson D., Howard R.E., Hubbard W., Jackel L.D. (1989). Backpropagation Applied to Handwritten Zip Code Recognition. Neural Comput..

[82] Long J., Shelhamer E., Darrell T. Fully convolutional networks for semantic segmentation. Proceedings of the IEEE Conference on Computer Vision and Pattern Recognition.

[83] Qin W., Wu J., Han F., Yuan Y., Zhao W., Ibragimov B., Gu J., Xing L. (2018). Superpixel-based and boundary-sensitive convolutional neural network for automated liver segmentation. Phys. Med. Biol..

[84] Gheshlaghi S.H., Ranjbar A., Suratgar A.A., Menhaj M.B., Faraji F. (2019). A superpixel segmentation based technique for multiple sclerosis lesion detection. arXiv.

[85] Fang L., Wang X., Wang M. (2021). Superpixel/voxel medical image segmentation algorithm based on the regional interlinked value. Pattern Anal. Appl..

[86] Ronneberger O., Fischer P., Brox T., Navab N., Hornegger J., Wells W.M., Frangi A.F. U-net: Convolutional networks for biomedical image segmentation. Proceedings of the International Conference on Medical Image Computing and Computer-Assisted Intervention.

[87] Goodfellow I.J., Pouget-Abadie J., Mirza M., Xu B., Warde-Farley D., Ozair S., Courville A., Bengio Y. (2014). Generative Adversarial Networks. arXiv.

[88] Radford A., Metz L., Chintala S. (2016). Unsupervised Representation Learning with Deep Convolutional Generative Adversarial Networks. arXiv.

[89] Jenni S., Favaro P. On Stabilizing Generative Adversarial Training With Noise. Proceedings of the 2019 IEEE/CVF Conference on Computer Vision and Pattern Recognition (CVPR).

[90] Mo S., Cho M., Shin J. (2020). Freeze the discriminator: A simple baseline for fine-tuning gans. arXiv.

[91] Hochreiter S., Schmidhuber J. (1997). Long short-term memory. Neural Comput..

[92] Gers F. Learning to forget: Continual prediction with LSTM. Proceedings of the 9th International Conference on Artificial Neural Networks: ICANN ’99.

[93] Gers F., Schmidhuber J. Recurrent nets that time and count. Proceedings of the IEEE-INNS-ENNS International Joint Conference on Neural Networks. IJCNN 2000. Neural Computing: New Challenges and Perspectives for the New Millennium.

[94] Cho K., van Merriënboer B., Gulcehre C., Bahdanau D., Bougares F., Schwenk H., Bengio Y. Learning Phrase Representations using RNN Encoder–Decoder for Statistical Machine Translation. Proceedings of the 2014 Conference on Empirical Methods in Natural Language Processing (EMNLP).

[95] Karimi Jafarbigloo S., Danyali H. (2021). Nuclear atypia grading in breast cancer histopathological images based on CNN feature extraction and LSTM classification. CAAI Trans. Intell. Technol..

[96] Azad R., Asadi-Aghbolaghi M., Fathy M., Escalera S. Bi-directional ConvLSTM U-Net with densley connected convolutions. Proceedings of the IEEE/CVF International Conference on Computer Vision Workshops.

[97] Scarselli F., Gori M., Tsoi A.C., Hagenbuchner M., Monfardini G. (2009). The Graph Neural Network Model. IEEE Trans. Neural Netw..

[98] Zhou J., Cui G., Hu S., Zhang Z., Yang C., Liu Z., Wang L., Li C., Sun M. (2020). Graph neural networks: A review of methods and applications. AI Open.

[99] Kipf T.N., Welling M. (2017). Semi-Supervised Classification with Graph Convolutional Networks. arXiv.

[100] Wu F., Souza A., Zhang T., Fifty C., Yu T., Weinberger K. Simplifying Graph Convolutional Networks. Proceedings of the 36th International Conference on Machine Learning.

[101] Zhou Y., Graham S., Alemi Koohbanani N., Shaban M., Heng P.A., Rajpoot N. CGC-Net: Cell Graph Convolutional Network for Grading of Colorectal Cancer Histology Images. Proceedings of the 2019 IEEE/CVF International Conference on Computer Vision Workshop (ICCVW).

[102] Yu K., Xie W., Wang L., Zhang S., Li W. (2021). Determination of biomarkers from microarray data using graph neural network and spectral clustering. Sci. Rep..

[103] Li W., Xie W., Zhang S., Wang L., Yang J., Zhao D. (2022). A Novel Biomarker Selection Method Combining Graph Neural Network and Gene Relationships Applied to Microarray Data. Preprint.

[104] Vaswani A., Shazeer N., Parmar N., Uszkoreit J., Jones L., Gomez A.N., Kaiser Ł., Polosukhin I. (2017). Attention is all you need. Adv. Neural Inf. Process. Syst..

[105] Bahdanau D., Cho K., Bengio Y. (2014). Neural machine translation by jointly learning to align and translate. arXiv.

[106] Parvaiz A., Khalid M.A., Zafar R., Ameer H., Ali M., Fraz M.M. (2022). Vision Transformers in Medical Computer Vision—A Contemplative Retrospection. arXiv.

[107] Dosovitskiy A., Beyer L., Kolesnikov A., Weissenborn D., Zhai X., Unterthiner T., Dehghani M., Minderer M., Heigold G., Gelly S. (2020). An image is worth 16x16 words: Transformers for image recognition at scale. arXiv.

[108] Chen J., Lu Y., Yu Q., Luo X., Adeli E., Wang Y., Lu L., Yuille A.L., Zhou Y. (2021). Transunet: Transformers make strong encoders for medical image segmentation. arXiv.

[109] Lum T., Mahdavi M., Frenkel O., Lee C., Jafari M.H., Dezaki F.T., Woudenberg N.V., Gu A.N., Abolmaesumi P., Tsang T. Imaging Biomarker Knowledge Transfer for Attention-Based Diagnosis of COVID-19 in Lung Ultrasound Videos. Proceedings of the International Workshop on Advances in Simplifying Medical Ultrasound.

[110] Lan E. (2022). Performer: A Novel PPG to ECG Reconstruction Transformer For a Digital Biomarker of Cardiovascular Disease Detection. arXiv.

[111] Oh S.J., Schiele B., Fritz M. (2019). Towards Reverse-Engineering Black-Box Neural Networks. Explainable AI: Interpreting, Explaining and Visualizing Deep Learning.

[112] Buhrmester V., Münch D., Arens M. (2021). Analysis of Explainers of Black Box Deep Neural Networks for Computer Vision: A Survey. Mach. Learn. Knowl. Extr..

[113] Starke G., De Clercq E., Elger B. (2021). Towards a pragmatist dealing with algorithmic bias in medical machine learning. Med. Health Care Philos..

[114] Tommasi T., Patricia N., Caputo B., Tuytelaars T., Csurka G. (2017). A Deeper Look at Dataset Bias. Domain Adaptation in Computer Vision Applications.

[115] Al-shayea Q.K. (2011). Artificial Neural Networks in Medical Diagnosis. Int. J. Comput. Sci. Issues (IJCSI).

[116] Amato F., López A., Peña-Méndez E.M., Vaňhara P., Hampl A., Havel J. (2013). Artificial neural networks in medical diagnosis. J. Appl. Biomed..

[117] Zherebtsov E., Dremin V., Popov A., Doronin A., Kurakina D., Kirillin M., Meglinski I., Bykov A. (2019). Hyperspectral imaging of human skin aided by artificial neural networks. Biomed. Opt. Express.

[118] Fredriksson I., Larsson M., Strömberg T. (2020). Machine learning for direct oxygen saturation and hemoglobin concentration assessment using diffuse reflectance spectroscopy. J. Biomed. Opt..

[119] Moncayo S., Manzoor S., Ugidos T., Navarro-Villoslada F., Caceres J. (2014). Discrimination of human bodies from bones and teeth remains by Laser Induced Breakdown Spectroscopy and Neural Networks. Spectrochim. Acta Part At. Spectrosc..

[120] Al-Hetlani E., Halámková L., Amin M.O., Lednev I.K. (2020). Differentiating smokers and nonsmokers based on Raman spectroscopy of oral fluid and advanced statistics for forensic applications. J. Biophotonics.

[121] Gao X.W., Hui R., Tian Z. (2017). Classification of CT brain images based on deep learning networks. Comput. Methods Programs Biomed..

[122] Yu-Jen Chen Y.J., Hua K.L., Hsu C.H., Cheng W.H., Hidayati S.C. (2015). Computer-aided classification of lung nodules on computed tomography images via deep learning technique. OncoTargets Ther..

[123] Saha M., Chakraborty C., Racoceanu D. (2018). Efficient deep learning model for mitosis detection using breast histopathology images. Comput. Med. Imaging Graph..

[124] Jafarbiglo S.K., Danyali H., Helfroush M.S. Nuclear atypia grading in histopathological images of breast cancer using convolutional neural networks. Proceedings of the 2018 4th Iranian Conference on Signal Processing and Intelligent Systems (ICSPIS).

[125] Chen M., Zhang B., Topatana W., Cao J., Zhu H., Juengpanich S., Mao Q., Yu H., Cai X. (2020). Classification and mutation prediction based on histopathology H&E images in liver cancer using deep learning. NPJ Precis. Oncol..

[126] Alex V., Safwan K.P.M., Chennamsetty S.S., Krishnamurthi G. (2017). Generative Adversarial Networks for Brain Lesion Detection. Med. Imaging.

[127] Son J., Park S.J., Jung K.H. (2017). Retinal vessel segmentation in fundoscopic images with generative adversarial networks. arXiv.

[128] Zhang Q., Wang H., Lu H., Won D., Yoon S.W. Medical Image Synthesis with Generative Adversarial Networks for Tissue Recognition. Proceedings of the 2018 IEEE International Conference on Healthcare Informatics (ICHI).

[129] Nie D., Trullo R., Lian J., Petitjean C., Ruan S., Wang Q., Shen D., Descoteaux M., Maier-Hein L., Franz A., Jannin P., Collins D.L., Duchesne S. (2017). Medical Image Synthesis with Context-Aware Generative Adversarial Networks. Proceedings of the Medical Image Computing and Computer Assisted Intervention—MICCAI 2017.

[130] Lipton Z.C., Berkowitz J., Elkan C. (2015). A Critical Review of Recurrent Neural Networks for Sequence Learning. arXiv.

[131] Futoma J., Hariharan S., Heller K., Sendak M., Brajer N., Clement M., Bedoya A., O’Brien C., Doshi-Velez F., Fackler J., Kale D., Ranganath R., Wallace B., Wiens J. (2017). An Improved Multi-Output Gaussian Process RNN with Real-Time Validation for Early Sepsis Detection. Proceedings of the 2nd Machine Learning for Healthcare Conference.

[132] Giunchiglia E., Nemchenko A., van der Schaar M., Kůrková V., Manolopoulos Y., Hammer B., Iliadis L., Maglogiannis I. (2018). RNN-SURV: A Deep Recurrent Model for Survival Analysis. Proceedings of the Artificial Neural Networks and Machine Learning—ICANN 2018.

[133] Reddy B.K., Delen D. (2018). Predicting hospital readmission for lupus patients: An RNN-LSTM-based deep-learning methodology. Comput. Biol. Med..

[134] Wang P., Chen E.Z., Chen T., Patel V.M., Sun S. (2020). Pyramid Convolutional RNN for MRI Reconstruction. IEEE Trans. Med. Imaging.

[135] Li Y., Qian B., Zhang X., Liu H. (2020). Graph Neural Network-Based Diagnosis Prediction. Big Data.

[136] Liu S., Li T., Ding H., Tang B., Wang X., Chen Q., Yan J., Zhou Y. (2020). A hybrid method of recurrent neural network and graph neural network for next-period prescription prediction. Int. J. Mach. Learn. Cybern..

[137] Li X., Dvornek N.C., Zhou Y., Zhuang J., Ventola P., Duncan J.S., Shen D., Liu T., Peters T.M., Staib L.H., Essert C., Zhou S., Yap P.T., Khan A. (2019). Graph Neural Network for Interpreting Task-fMRI Biomarkers. Medical Image Computing and Computer Assisted Intervention—MICCAI 2019.

[138] Shi J., Wang R., Zheng Y., Jiang Z., Zhang H., Yu L. (2021). Cervical cell classification with graph convolutional network. Comput. Methods Programs Biomed..

[139] Steinkraus D., Buck I., Simard P. Using GPUs for machine learning algorithms. Proceedings of the Eighth International Conference on Document Analysis and Recognition (ICDAR’05).

